# Severe tricuspid regurgitation: aetiology, patient characteristics, and treatment: a multicentre, prospective registry

**DOI:** 10.1093/eschf/xvag190

**Published:** 2026-07-16

**Authors:** Agnieszka Kapłon-Cieślicka, Adam Piasecki, Karolina Semczuk-Kaczmarek, Katarzyna Holcman, Sylwia Szczepara, Grzegorz Kopeć, Agata Krawczyk-Ożóg, Renata Rajtar-Salwa, Danuta Sorysz, Karol Kamiński, Małgorzata Knapp, Sebastian Stelmaszek, Maria Królikowska, Anna Furman-Niedziejko, Andrzej Gackowski, Agata Polańska-Szczap, Grażyna Tuskowska, Marek Koziński, Anna Tomaszuk-Kazberuk, Kinga Dudzińska, Paweł Muszyński, Ludmiła Daniłowicz-Szymanowicz, Damian Kaufmann, Małgorzata Szwoch, Beata Uziębło-Życzkowska, Małgorzata Maciorowska, Paweł Krzesiński, Agata Markiewicz, Agnieszka Bartczak-Rutkowska, Marek Grygier, Bartosz Kijewski, Robert Błaszczyk, Bartosz Kondracki, Piotr Nikodem Rudziński, Marcin Demkow, Mariusz Dębski, Tomasz Tokarek, Dominika Dykla, Aleksander Zeliaś, Rafał Gałąska, Marcin Fijałkowski, Małgorzata Niemiec, Katarzyna Mizia-Stec, Katarzyna Kurnicka, Piotr Pruszczyk, Jerzy Pręgowski, Jarosław Skowroński, Agata Bielecka-Dąbrowa, Karolina Kupczyńska, Ewa Pędzich, Ewa Ostrowska, Mariusz Tomaniak, Marcin Grabowski, Piotr Scisło, Adam Rdzanek

**Affiliations:** 1st Chair and Department of Cardiology, Medical University of Warsaw, Banacha 1A, Warsaw 02-097, Poland; 1st Chair and Department of Cardiology, Medical University of Warsaw, Banacha 1A, Warsaw 02-097, Poland; 1st Chair and Department of Cardiology, Medical University of Warsaw, Banacha 1A, Warsaw 02-097, Poland; Department of Cardiac and Vascular Diseases, Jagiellonian University Medical College, Institute of Cardiology, St. John Paul II Hospital, Kraków, Poland; Department of Nuclear Medicine, St. John Paul II Hospital, Kraków, Poland; Department of Cardiac and Vascular Diseases, Jagiellonian University Medical College, Institute of Cardiology, St. John Paul II Hospital, Kraków, Poland; Department of Cardiac and Vascular Diseases, Jagiellonian University Medical College, Institute of Cardiology, St. John Paul II Hospital, Kraków, Poland; Pulmonary Circulation Centre Department of Cardiac and Vascular Diseases, Jagiellonian University Medical College, Kraków, Poland; Department of Cardiology and Cardiovascular Interventions, University Hospital, Kraków, Poland; Department of Anatomy, Jagiellonian University Medical College, Kraków, Poland; Department of Cardiology and Cardiovascular Interventions, University Hospital, Kraków, Poland; 2nd Department of Cardiology, Jagiellonian University Medical College, Kraków, Poland; Department of Cardiology and Cardiovascular Interventions, University Hospital, Kraków, Poland; 2nd Department of Cardiology, Jagiellonian University Medical College, Kraków, Poland; Department of Cardiology and Internal Medicine with Cardiac Intensive Care Unit, Medical University of Bialystok, Bialystok, Poland; Department of Population Medicine and Prevention of Lifestyle Diseases, Medical University of Bialystok, Bialystok, Poland; Department of Cardiology and Internal Medicine with Cardiac Intensive Care Unit, Medical University of Bialystok, Bialystok, Poland; Department of Cardiology and Internal Medicine with Cardiac Intensive Care Unit, Medical University of Bialystok, Bialystok, Poland; Department of Coronary Disease and Heart Failure, Jagiellonian University, Medical College, St. John Paul II Hospital, Kraków, Poland; Department of Coronary Disease and Heart Failure, Jagiellonian University, Medical College, St. John Paul II Hospital, Kraków, Poland; Department of Emergency Medicine, Faculty of Health Sciences, Jagiellonian University Medical College, Kraków, Poland; Department of Coronary Disease and Heart Failure, Jagiellonian University, Medical College, St. John Paul II Hospital, Kraków, Poland; Department of Cardiology, Kociewie Health Center, Starograd Gdanski, Poland; Department of Cardiology, Kociewie Health Center, Starograd Gdanski, Poland; Department of Cardiology, Kociewie Health Center, Starograd Gdanski, Poland; 1st Department of Cardiology, Medical University of Gdansk, Gdansk, Poland; Department of Cardiology, Lipidology and Internal Medicine with Intensive Cardiac Care Unit, Medical University of Bialystok, Bialystok, Poland; Department of Cardiology, Lipidology and Internal Medicine with Intensive Cardiac Care Unit, Medical University of Bialystok, Bialystok, Poland; Department of Cardiology, Lipidology and Internal Medicine with Intensive Cardiac Care Unit, Medical University of Bialystok, Bialystok, Poland; Department of Cardiology and Electrotherapy, Faculty of Medicine, Medical University of Gdansk, Gdansk, Poland; Department of Cardiology and Electrotherapy, Faculty of Medicine, Medical University of Gdansk, Gdansk, Poland; Department of Cardiology and Electrotherapy, Faculty of Medicine, Medical University of Gdansk, Gdansk, Poland; Department of Cardiology and Internal Diseases, Military Institute of Medicine—National Research Institute, Warsaw, Poland; Department of Cardiology and Internal Diseases, Military Institute of Medicine—National Research Institute, Warsaw, Poland; Department of Cardiology and Internal Diseases, Military Institute of Medicine—National Research Institute, Warsaw, Poland; 1st Department of Cardiology, Poznan University of Medical Sciences, Poznan, Poland; 1st Department of Cardiology, Poznan University of Medical Sciences, Poznan, Poland; 1st Department of Cardiology, Poznan University of Medical Sciences, Poznan, Poland; Department of Cardiology, Medical University of Lublin, University Centre of Cardiology and Cardiac Surgery, Lublin, Poland; Department of Cardiology, Medical University of Lublin, University Centre of Cardiology and Cardiac Surgery, Lublin, Poland; Department of Cardiology, Medical University of Lublin, University Centre of Cardiology and Cardiac Surgery, Lublin, Poland; Department of Coronary and Structural Heart Diseases, The Cardinal Stefan Wyszyński National Institute of Cardiology, Warsaw, Poland; Department of Coronary and Structural Heart Diseases, The Cardinal Stefan Wyszyński National Institute of Cardiology, Warsaw, Poland; Department of Coronary and Structural Heart Diseases, The Cardinal Stefan Wyszyński National Institute of Cardiology, Warsaw, Poland; 2nd Clinical Department of Cardiology, Faculty of Medicine and Health Science, University of Applied Science in Nowy Sącz, Nowy Sącz, Poland; Center for Innovative Medical Education, Jagiellonian University Medical College, Kraków, Poland; 2nd Clinical Department of Cardiology, Faculty of Medicine and Health Science, University of Applied Science in Nowy Sącz, Nowy Sącz, Poland; The Faculty of Medicine and Health Sciences, University of Applied Sciences in Nowy Sącz, Nowy Sącz, Poland; 2nd Clinical Department of Cardiology, Faculty of Medicine and Health Science, University of Applied Science in Nowy Sącz, Nowy Sącz, Poland; The Faculty of Medicine and Health Sciences, University of Applied Sciences in Nowy Sącz, Nowy Sącz, Poland; 1st Department of Cardiology, Medical University of Gdansk, Gdansk, Poland; 1st Department of Cardiology, Medical University of Gdansk, Gdansk, Poland; First Department of Cardiology, Chair of Cardiology, School of Medicine in Katowice, Medical University of Silesia, Katowice, Poland; Centre of the European Reference Network for Rare, Low Prevalence or Complex Diseases of the Heart (ERN GUARD Heart), Katowice, Poland; First Department of Cardiology, Chair of Cardiology, School of Medicine in Katowice, Medical University of Silesia, Katowice, Poland; Centre of the European Reference Network for Rare, Low Prevalence or Complex Diseases of the Heart (ERN GUARD Heart), Katowice, Poland; Independent Scientific Echocardiography Laboratory, National Institute of Geriatrics, Rheumatology and Rehabilitation, Warsaw, Poland; Department of Internal Medicine and Cardiology, Medical University of Warsaw, Warsaw, Poland; Department of Interventional Cardiology and Angiology, National Institute of Cardiology, Warsaw, Poland; Department of Interventional Cardiology and Angiology, National Institute of Cardiology, Warsaw, Poland; Department of Preventive Cardiology and Lipidology, Medical University of Lodz, Lodz, Poland; Department of Cardiology and Congenital Heart Diseases of Adults, Polish Mother's Memorial Hospital Research Institute (PMMHRI), Lodz, Poland; Department of Cardiology and Congenital Heart Diseases of Adults, Polish Mother's Memorial Hospital Research Institute (PMMHRI), Lodz, Poland; 1st Chair and Department of Cardiology, Medical University of Warsaw, Banacha 1A, Warsaw 02-097, Poland; 1st Chair and Department of Cardiology, Medical University of Warsaw, Banacha 1A, Warsaw 02-097, Poland; 1st Chair and Department of Cardiology, Medical University of Warsaw, Banacha 1A, Warsaw 02-097, Poland; 1st Chair and Department of Cardiology, Medical University of Warsaw, Banacha 1A, Warsaw 02-097, Poland; 1st Chair and Department of Cardiology, Medical University of Warsaw, Banacha 1A, Warsaw 02-097, Poland; 1st Chair and Department of Cardiology, Medical University of Warsaw, Banacha 1A, Warsaw 02-097, Poland

**Keywords:** Tricuspid valve, Right-sided heart failure, Transcatheter edge-to-edge repair, Surgery, Lead extraction

## Abstract

**Background and Aims:**

Severe tricuspid regurgitation (TR) is associated with increased mortality and hospitalizations for heart failure (HF). Aetiologies of TR include: secondary (atrial or ventricular), primary and cardiac implantable electronic device (CIED)-related. The aim of the study was to assess the prevalence of different TR aetiologies, as well as characteristics and treatment of patients with severe TR in a real-life setting.

**Methods:**

This was a prospective, observational study of patients with severe TR, conducted in 18 cardiology centres. Consecutive adult patients with severe TR were included, regardless of the presence of TR symptoms and the cause of hospital admission.

**Results:**

A total of 1295 patients with severe TR were enrolled (median age 76 years, 53% women). The most common reason for admission was HF decompensation (40%). Single TR aetiology was identified in 79% patients. The most frequent overlap between aetiologies included secondary ventricular and atrial TR, and was found in 11% of patients. The most common TR aetiology was secondary atrial (37%), followed by secondary ventricular (25%). Primary and CIED-related TR accounted for 10% and 15%, respectively. In 2.4% TR aetiology was not determined. Patients with secondary atrial and CIED-related TR were the oldest (79 and 78 years, respectively), and those with primary TR the youngest (68 years). Patients with CIED-related and secondary ventricular TR were more often hospitalized for HF decompensation, had more advanced HF symptoms, worse left- and right-ventricular function, worse kidney and liver function, and higher in-hospital mortality. Only 40% of patients underwent evaluation by the Heart Team and 21% were qualified for TR interventions.

**Conclusions:**

In real life, unequivocal identification of TR aetiology remains challenging. Secondary atrial TR is the most common aetiology. There are significant differences in characteristics and outcomes depending on TR aetiology. Too few patients with severe TR undergo evaluation by the Heart Team.

## Graphical Abstract

**Graphical Abstract:**
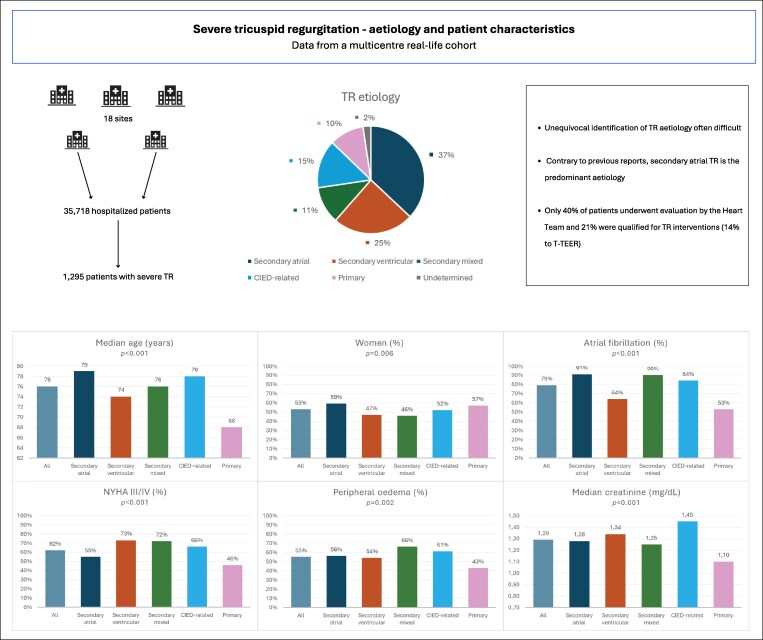

## Introduction

Tricuspid regurgitation (TR) remains one of the most common valvular diseases, with significant TR (moderate or severe) affecting ∼4% of people aged 75 years or more.^1–3^ Severe TR is associated with frequent hospitalizations for heart failure (HF) and increased mortality.^4–6^ In fact, among patients with HF, those with isolated, significant TR seem to be at a higher risk for HF hospitalization than those with isolated, significant mitral regurgitation or even those with combined mitral and TR.^7–9^ Due to the anatomy of the tricuspid valve, which is the biggest valve in the human body and has very thin and delicate leaflets, as well as the potential of the right ventricle, right atrium and the tricuspid annulus to dilate extensively with volume overload, TR can become extremely severe, which prompted the development of nomenclature to describe more advance grades of severe TR, i.e. massive and torrential.^10^ Thus, TR is the only valvular disease with not 3 but 5 grades of severity, enabling accurate description of the disease stage, as well as the magnitude of TR reduction during transcatheter procedures. Introduction of those procedures and a growing interest in this once ‘forgotten’ valve have led to improved understanding of its complex anatomy and function, as well as TR aetiology. Given that TR treatment options depend on the underlying pathology, the most recent 2025 European Society of Cardiology (ESC) guidelines on valvular heart disease underline the need to identify TR aetiology: primary, secondary (ventricular or atrial), and cardiac implantable electronic device (CIED)-related.^1,10^ However, in clinical practice, a clear distinction between TR aetiologies may sometimes be challenging and overlap may occur. Furthermore, according to current literature, atrial secondary TR accounts for 10%–15% of clinically relevant TRs,^11,12^ but everyday clinical observation suggests higher prevalence, especially in older patients. The aim of this study was to assess the prevalence of different TR aetiologies, as well as characteristics and treatment of patients with severe TR in a real-life setting.

## Methods

### Study design

This was a multicentre, prospective, observational study of patients with severe TR, conducted in 18 cardiology centres. The study enrolled consecutive adult patients with severe TR, admitted to cardiology departments during the recruitment period. All consecutive patients with severe TR were included regardless of the presence of TR symptoms and regardless of the cause of hospital admission (both urgent and elective admissions, including hospitalizations for HF decompensation, atrial fibrillation, acute coronary syndromes, pulmonary embolism, infective endocarditis, or any other cause, as well as for elective diagnostic and therapeutic procedures, including assessment of eligibility for interventional treatment of valvular disease, coronary angiography, CIED implantation, exchange or extraction, etc.). Inclusion criterion was presence of severe TR at any time point during hospitalization (including patients in whom TR was reduced during hospitalization).

Patient recruitment process started in July 2024 in the co-ordinating centre and lasted 6 months since the beginning of the study in each participating centre or longer, i.e. until the inclusion of at least 30 patients at each participating centre (with last patients recruited in July 2025). Patients who were hospitalized multiple times during the study period were entered in the database under the same number.

Data were gathered prospectively and included demographics, medical history, pharmacotherapy, and results of routine laboratory tests. The assessment of both TR severity (1. mild or trace or absent, 2. moderate, 3. severe, 4. massive, and 5. torrential) and TR aetiology (1. primary, 2. secondary ventricular, 3. secondary atrial, and 4. CIED-related—with the possibility to choose more than one aetiology) were left at the discretion of investigators in each participating centre. The possible aetiologies were defined according to the current guidelines and expert consensus as follows.^13^ Primary TR was identified in the presence of clear anatomical abnormalities or degeneration of the tricuspid valve apparatus, such as leaflet prolapse or flail, congenital displacement of leaflets or TV apparatus or leaflet or chordae injury. CIED-related TR was identified in the presence of a causative relationship between right-ventricular lead and TR resulting from an interaction of RV lead with TV apparatus, such as leaflet impingement, perforation, adherence or interference with leaflet motion. In the absence of structural abnormalities of TV, secondary TR was identified and categorized as secondary ventricular or secondary atrial. Secondary ventricular TR was defined as leaflet tethering resulting from right-ventricular dilatation and/or dysfunction, while secondary atrial TR was defined as resulting from tricuspid annular dilatation (due to right-atrial dilatation) with minimal or no leaflet tethering. Investigators were asked to identify one, most relevant TR aetiology. There was no core laboratory evaluation or validation of echocardiographic data. If an investigator denoted more than one possible TR aetiologies, the following criteria were applied to categorize patients: when ‘CIED-related’ was one of the denoted aetiologies, TR was categorized as CIED-related; when ‘primary’ was one of the denoted aetiologies, unless ‘CIED-related’ was also chosen—TR aetiology was categorized as primary. Patients in whom both ‘secondary ventricular’ and ‘secondary atrial’ were denoted aetiologies, were categorized in a distinct category ‘secondary mixed’.^11^

If control echocardiography was performed during hospitalization, the result was also entered in the database. No long-term follow-up was planned within the registry but if a patient was readmitted during the study period, this fact (along with the reason for readmission) was recorded in the database.

The study was approved by the Ethics Committee of Medical University of Warsaw (AKBE/39/2023). Data were entered into the registry database anonymously. No additional tests or interventions, apart from those planned by the attending physicians, were performed. Thus, the ethics committee waived the requirement of obtaining informed consent from the patients. Patients and public have not been involved in the design, conduct or reporting of this research.

### Statistical analysis

Statistical analysis was conducted using IBM SPSS Statistics version 29.0 (IBM, Armonk, NY, USA). Categorical variables are presented as number and percentage. Continuous variables are presented as median and inter-quartile range (IQR) or mean and standard deviation (SD) depending on the distribution. Normality of the distribution was tested using Shapiro–Wilk test. Initial comparisons included five groups of patients categorized by TR aetiology. Categorical variables were compared between groups using χ^2^ test. The difference in distribution of the continuous variables between the groups was assessed with Kruskal–Wallis test. For intra-group comparisons of categorical variables of any two groups χ^2^ test was used. Student’s *t*-test or *U* Mann–Whitney test was used to compare the distribution of any two groups of continuous variables depending on the normality of distribution. A *P*-value of <.05 was considered statistically significant. All presented *P*-values are two-sided.

## Results

In the registry, 1295 patients with severe TR were enrolled in 18 centres (16 tertiary, 1 secondary, and 1 primary [first-level] hospital). During the study period, all those cardiology departments hospitalized a total of 35 718 patients, with patients with severe TR constituting 3.6% of all hospitalized patients.

Median age of patients with severe TR was 76 years (IQR 69–83 years), and 53% were women. The most common primary reason for admission was HF decompensation (40%). Other reasons included: elective hospitalization for the assessment of eligibility for valvular intervention (27%), atrial fibrillation (9.3%), chronic coronary syndrome (5.9%), CIED implantation, exchange or extraction (5.3%), acute coronary syndrome (3.2%), pulmonary embolism (1.5%), infective endocarditis (1.0%), and other causes (26%).

Majority of patients (98%) had a native tricuspid valve, 21 patients (1.6%) were after surgical tricuspid valve repair, 5 (0.4%) after surgical tricuspid valve replacement, and 7 (0.5%) after transcatheter tricuspid intervention (including tricuspid transcatheter edge-to-edge repair in 6 patients). On admission, CIEDs were present in 421 (33%) patients. The most advanced grade of TR during hospitalization was categorized as severe in 942 (73%), massive in 221 (17%), and torrential in 132 (10%) patients.

TR aetiology was reported by investigators with the possibility to choose more than one fitting cause. Single aetiology was identified in 1022 (79%) patients. In 242 (19%) patients, two or three TR aetiologies were chosen by the investigators. However, in 31 (2.4%) cases, TR aetiology was not reported by the investigator. *Table 1* presents the proportion of patients with different TR aetiologies identified by investigators. Compared with tertiary centres, the two lower-level centres more often identified single TR aetiology (78% vs 89%, *P* = .002).

**Table 1:** Number and proportion of patients with different TR aetiologies as denoted by investigators (more than one aetiology could be designated for one patient)

Tricuspid regurgitation aetiology in 1295 patients	*n* (%)
**Single aetiology**	**1022** (**79**)
Secondary atrial	479 (37)
Secondary ventricular	317 (25)
CIED	115 (8.9)
Primary	111 (8.6)
**Double aetiology**	**225** (**17)**
Secondary atrial + secondary ventricular	145 (11)
Secondary atrial + CIED	35 (2.7)
Secondary atrial + primary	16 (1.2)
Secondary ventricular + CIED	22 (1.7)
Secondary ventricular + primary	6 (0.5)
CIED + primary	1 (0.1)
**Triple aetiology**	**17** (**1.3)**
Secondary atrial + ventricular + CIED	12 (0.9)
Secondary atrial + CIED + primary	2 (0.2)
Secondary ventricular + CIED + primary	1 (0.1)
Secondary atrial + secondary ventricular + primary	2 (0.2)
**Undetermined**	**31** (**2.4)**

CIED, cardiac implantable electronic device

**Figure 1:**
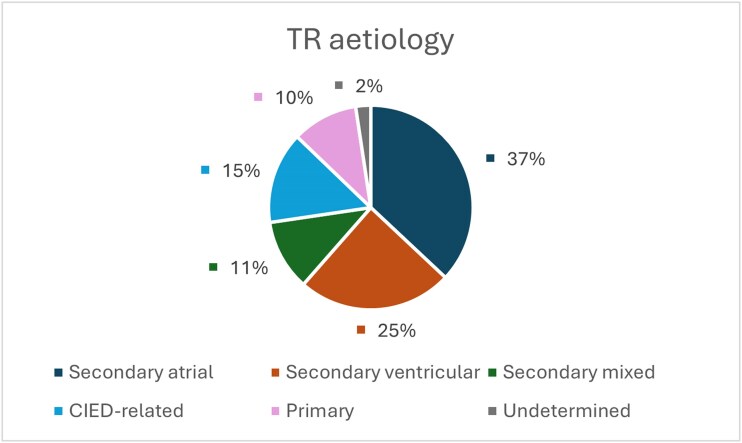
Predominant TR aetiologies. TR, tricuspid regurgitation; CIED, cardiac implantable electronic device

Finally, following the criteria described above, 188 (15%) patients were categorized as having predominantly CIED-related TR, 135 (10%)—predominantly primary TR, 479 (37%)—secondary atrial TR, 317 (25%)—secondary ventricular TR, 145 (11%)—secondary mixed (ventricular and atrial) TR, and 31 (2.4%) TR of undetermined aetiology (*Figure 1*).

There were some significant differences between the 5 aetiology groups (secondary atrial, secondary ventricular, secondary mixed, CIED-related and primary TR) with regard to demographics, clinical and laboratory characteristics, as well as to treatment and in-hospital outcomes (*Table 2*).

**Table 2:** Characteristics and outcomes of patients with severe TR in relation to dominant aetiology

	All patients (*n* = 1295)	Secondary atrial TR (*n* = 479)	Secondary ventricular TR (*n* = 317)	Secondary mixed (atrial + ventricular) TR (*n* = 145)	CIED-related TR (*n* = 188)	Primary TR (*n* = 135)	*P*
Age, years, median (IQR)	76.0 (69.0–83.0)	79.0 (73.0–85.0)	74.0 (66.0–80.0)	76.0 (70.0–83.0)	78.0 (71.0–84.0)	68.0 (43.0–77.0)	<.001
Women, *n* (%)	687 (53)	280 (59)	149 (47)	66 (46)	98 (52)	77 (57)	.006
Hospitalization for HF decompensation, *n* (%)	517 (40)	187 (39)	146 (46)	50 (35)	83 (44)	34 (25)	<.001
TR *de novo*, *n* (%)	497 (38)	209 (44)	133 (42)	53 (37)	48 (26)	34 (25)	<.001
TR diagnosed >12 months before index hospitalization, *n* (%)	470 (36)	164 (34)	95 (30)	52 (36)	88 (47)	66 (59)	<.001
TR grade (most severe during hospitalization), *n* (%)							<.001
**Severe**	942 (73)	354 (74)	253 (80)	94 (65)	116 (62)	26 (84)	
**Massive**	221 (17)	87 (18)	39 (12)	33 (23)	44 (23)	16 (12)	
**Torrential**	132 (10)	38 (7.9)	25 (7.9)	18 (12)	28 (15)	20 (15)	
Medical history							
Atrial fibrillation, *n* (%)	1019 (79)	435 (91)	203 (64)	131 (90)	159 (84)	71 (53)	<.001
**Permanent AF**	700 (54)	323 (67)	125 (39)	92 (63)	110 (59)	39 (29)	<.001
HF, *n* (%)	134 (88)	429 (90)	288 (91)	119 (82)	172 (92)	98 (73)	<.001
**HFrEF**	385 (30)	97 (23)	131 (46)	45 (38)	82 (48)	18 (18)	
**HFmrEF**	181 (14)	74 (17)	37 (13)	21 (13)	30 (17)	14 (14)	
**HFpEF**	567 (44)	258 (60)	119 (42)	53 (45)	60 (35)	66 (67)	<.001
Ischaemic HF aetiology, *n* (%)	455 (40)	149 (35)	139 (48)	50 (42)	78 (45)	28 (29)	<.001
Previous surgical treatment of left-sided valvular disease, *n* (%)	144 (11)	41 (8.6)	31 (9.8)	16 (11)	21 (11)	13 (9.6)	.831
Previous TAVR, *n* (%)	28 (2.2)	8 (1.7)	4 (1.3)	2 (1.4)	8 (4.3)	4 (3.0)	.150
Previous transcatheter treatment of mitral regurgitation, *n* (%)	34 (2.6)	6 (1.3)	8 (2.5)	2 (1.4)	9 (4.8)	6 (4.4)	.039
Significant mitral valve disease (currently), *n* (%)	288 (21)	84 (18)	83 (26)	38 (26)	40 (21)	14 (10)	<.001
**Severe secondary mitral regurgitation**	185 (14)	58 (12)	55 (17)	32 (22)	28 (15)	4 (3.0)	<.001
**Severe primary mitral regurgitation**	60 (4.6)	15 (3.1)	18 (5.7)	4 (2.8)	10 (5.3)	9 (6.7)	.201
**Significant (severe or moderate) mitral stenosis**	32 (2.5)	11 (2.3)	10 (3.2)	2 (1.4)	2 (1.1)	1 (0.7)	.349
Severe aortic valve disease—stenosis or regurgitation (currently), *n* (%)	62 (4.8)	14 (2.9)	20 (6.3)	7 (4.8)	13 (6.9)	5 (3.7)	.107
Pulmonary hypertension, *n* (%)	328 (25)	114 (24)	105 (33)	29 (20)	58 (31)	19 (14)	<.001
Chronic kidney disease (G3–G5), *n* (%)	526 (41)	196 (41)	132 (42)	59 (41)	87 (46)	41 (30)	.075
COPD or other chronic pulmonary disease, *n* (%)	229 (18)	79 (17)	67 (21)	23 (16)	25 (13)	16 (12)	.077
Liver disease, *n* (%)	50 (3.9)	13 (2.7)	16 (5.0)	9 (6.2)	8 (4.3)	4 (3.0)	.261
Diabetes, *n* (%)	377 (29)	146 (31)	96 (30)	41 (28)	66 (35)	21 (15)	.003
Symptoms on admission							
NYHA III/IV, *n* (%)	801 (62)	263 (55)	230 (73)	104 (72)	124 (66)	62 (46)	<.001
Peripheral oedema, *n* (%)	717 (55)	267 (56)	170 (54)	95 (66)	114 (61)	58 (43)	.002
Ascites, *n* (%)	134 (10)	45 (9.4)	37 (12)	17 (12)	21 (11)	13 (9.6)	.824
Pleural effusion, *n* (%)	239 (19)	79 (17)	85 (27)	27 (19)	34 (18)	11 (8.1)	<.001
Pericardial effusion, *n* (%)	234 (19)	90 (20)	71 (23)	21 (15)	26 (15)	19 (15)	.083
Laboratory tests							
NT-proBNP (highest), pg/ml (IQR)	3138 (1437–7614)	3036 (1628–6237)	4397 (1824–14019)	3252 (1360–7290)	3432(1676–7240)	1135 (307–3372)	<.001
Creatinine (highest), mg/dl (IQR)	1.29 (1.00–1.80)	1.28 (1.00–1.74)	1.34 (1.06–1.80)	1.25 (0.97–1.73)	1.45 (1.08–2.07)	1.10 (0.85–1.49)	<.001
eGFR (lowest) ml/min/1.73 m^2^ (IQR)	46.1 (31.0–60.0)	44.0 (30.0–60.0)	46.0 (32.0–60.0)	50.0 (37.0–60.0)	41.0 (28.0–57.2)	60.0 (40.0–60.0)	<.001
Bilirubin (highest) mg/dl (IQR)^a^	1.10 (0.70–1.85)	1.00 (0.62–1.85)	1.30 (0.80–2.70)	1.10 (0.67–1.74)	1.20 (0.80–1.90)	1.09 (0.71–1.59)	.004
Sodium (lowest), mmol/L (IQR)	138.0 (135.0–140.0)	138.0 (135.0–140.0)	138.0 (135.0–140.0)	139.0 (135.0–141.0)	138.0 (135.0–140.0)	139.0 (136.0–140.0)	.339
Echocardiography							
LVEF, % (IQR)	50.0 (37.0–58.0)	53.0 (45.0–60.0)	48.0 (30.0–55.0)	50.0 (33.0–57.5)	45.0 (28.0–55.0)	57.0 (50.0–60.0)	<.001
RVIT, mm (IQR)^b^	48.0 (42.0–54.0)	46.0 (41.0–52.0)	49.0 (43.0–55.0)	50.5 (46.0–56.0)	49.0 (43.0–53.0)	48.0 (42.0–56.0)	<.001
RAA, cm^2^ (IQR)	32.0 (26.0–40.0)	33.0 (27.0–41.0)	30.0 (24.2–37.0)	34.0 (27.2–40.0)	33.0 (26.0–40.8)	29.6 (25.0–38.0)	.001
TAPSE, mm (IQR)^c^	17.0 (14.0–20.0)	18.0 (15.0–20.0)	16.0 (13.0–19.0)	16.0 (13.0–19.0)	16.0 (14.0–20.0)	19.0 (15.0–22.0)	<.001
Treatment and outcomes							
Diuretic i.v. treatment during hospitalization, *n* (%)	620 (48)	221 (46)	180 (57)	70 (48)	92 (49)	35 (26)	<.001
In-hospital death, *n* (%)	58 (4.5)	14 (2.9)	22 (6.9)	2 (1.4)	14 (7.4)	2 (1.5)	.001
Patients qualified for an intervention^d^ by the Heart Team, *n* (%):	272 (21)	102 (21)	45 (14)	37 (26)	45 (24)	42 (31)	<.001
**Surgical tricuspid repair or replacement, *n* (%)**	59 (4.6)	19 (4.0)	13 (4.1)	7 (4.8)	7 (3.7)	13 (9.6)	.072
**Transcatheter tricuspid intervention, *n* (%)**	206 (16)	82 (17)	32 (10)	30 (21)	32 (17)	29 (22)	.007
Patients deemed ineligible for any procedure^d^ by the Heart Team, *n* (%)	90 (6.9)	23 (4.8)	24 (7.6)	12 (8.3)	11 (5.9)	16 (11.9)	.049
Patients not evaluated by the Heart Team, *n* (%)	782 (60)	306 (64)	212 (67)	78 (54)	105 (56)	58 (43)	<.001

AF, atrial fibrillation; ALT, alanine transaminase; COPD, chronic obstructive pulmonary disease; eGFR, estimated glomerular filtration rate; HFmrEF, heart failure with mildly reduced ejection fraction; HFpEF, heart failure with preserved ejection fraction; HFrEF, heart failure with reduced ejection fraction; IQR, inter-quartile range; LVEF, left-ventricular ejection fraction; NYHA, New York Heart Association; NT-proBNP, N-terminal pro-B-type natriuretic peptide; RAA, right-atrial area; RVIT, right-ventricular inflow tract; TAPSE, tricuspid annular plane systolic motion; TAVR, transcatheter aortic valve replacement; TR, tricuspid regurgitation.

^a^Data for 670 (52%) of all patients.

^b^Data for 1047 (81%) of all patients.

^c^Data for 1090 (84%) of all patients.

^d^Either tricuspid intervention (surgical or transcatheter) or lead extraction (surgical or percutaneous).

During hospitalization, intravenous diuretic treatment was administered in 620 (48%) of patients. Median weight reduction during hospitalization was 6.0 kg (IQR 4.0–9.3 kg). Echocardiography during hospitalization was repeated in 531 (41%) patients, showing a TR reduction to mild/trace/absent and moderate in 37 and 85 patients, respectively (altogether a reduction to mild/trace/absent or moderate TR in 122 [9.4%] of all patients). Out of 531 patients with repeated echocardiography, 317 had severe, 53—massive and 39—torrential TR.

**Figure 2:**
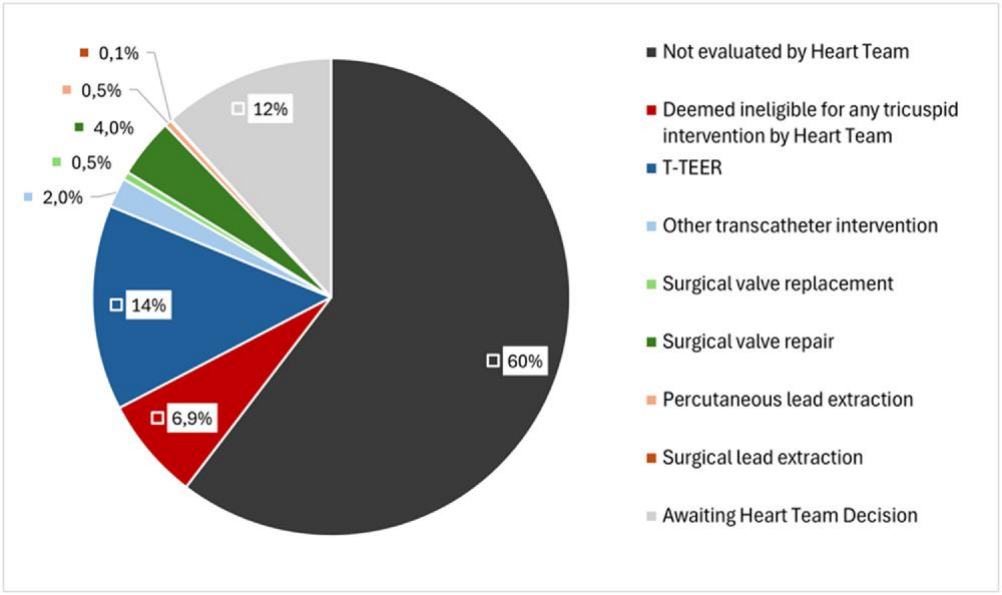
Interventional treatment of severe tricuspid regurgitation in 1295 patients included in the registry. T-TEER, tricuspid transcatheter edge-to-edge repair

Only 513 (40%) patients were evaluated for eligibility for tricuspid intervention by the Heart Team. The proportion of patients evaluated by the Heart Team was significantly lower in lower-level centres compared with tertiary centres (26% vs 41%, *P* < .001). Out of all 1295 patients, 90 (6.9%) patients were deemed ineligible for any tricuspid intervention by the Heart Team, 272 (21%) were qualified for an intervention for TR (97 had undergone the procedure during index hospitalization, and 175 were awaiting the procedure), and 151 (12%) were awaiting Heart Team decision. Of the 272 patients qualified for an intervention, 180 (66%) were qualified for tricuspid transcatheter edge-to-edge repair (TEER), 26 (9.6%) for a transcatheter tricuspid intervention other than TEER, 52 (19%) for surgical tricuspid valve repair, 7 (2.6%) for surgical tricuspid valve replacement, 6 patients for percutaneous lead extraction, and 1 patient for surgical lead extraction (*Figure 2*).

Fifty-eight (4.5%) patients died during hospitalization. Of those who survived, at discharge 72% received torasemide (at a median dose of 20 mg per day, IQR 10–20 mg), 39%—furosemide (median dose 80 mg per day, IQR 40–120 mg), 34%—eplerenone (median dose 25 mg per day, IQR 25–50 mg), 22% spironolactone (median dose 25 mg per day, IQR 25–50 mg), and 7.2%—hydrochlorothiazide (median dose 12.5 mg per day, IQR 12.5–25 mg).

## Discussion

The main outcome of the study is that an unequivocal identification of TR aetiology in real-life population was possible only for 79% and one in five patients had two or three possible pathomechanisms identified by the investigators with the biggest overlap between secondary atrial and secondary ventricular TR. This is most probably a result of a vicious circle due to right-ventricular and right-atrial volume overload in severe TR leading to right-ventricular enlargement and dysfunction in secondary atrial TR, and to right-atrial enlargement and annulus dilation in secondary ventricular TR.^11,14^ Thus, in advanced stages, a definite distinction between secondary atrial and ventricular TR may be challenging. Those patients, denoted as ‘secondary mixed’ TR, constituted a substantial proportion of patients (11%) and represented the most common overlap between TR aetiologies in our study.

Patients with secondary mixed TR shared some characteristics with either secondary atrial (proportion of patients with atrial fibrillation, NT-proBNP concentrations) or secondary ventricular (sex, tricuspid annular plane systolic motion [TAPSE]) TR or were ‘in-between’ those two groups with regard to some parameters (age, left-ventricular ejection fraction [LVEF], ischaemic aetiology of HF), but seemed a distinct group with regards to some other characteristics, showing, indeed, a more advanced stage of the ‘vicious circle’ compared with ‘pure’ atrial and ‘pure’ ventricular TR (e.g. in the ‘mixed’ group, TR was more often diagnosed more than 12 months ago, more often massive or torrential, associated with larger right ventricle and right atrium, and more often accompanied by severe secondary mitral regurgitation and liver disease compared with other secondary TR types). On the other hand, in secondary mixed TR, HF decompensation was less frequent the reason for current hospitalization and, consequently, was less frequently associated with in-hospital death compared with other types of secondary TR. This might be attributable to an inclusion bias as well as a relatively small number of patients in the secondary mixed group or might indicate that this group is, in fact, even more heterogeneous.

A novel observation in our study is that secondary atrial TR was found to be the most prevalent aetiology of severe TR, with ‘pure’ secondary atrial TR constituting 37% of all severe TRs. If analysed together with the secondary ‘mixed’ group (11% of all severe TR patients in our study), atrial TR would account for almost half of all severe TRs. This is contrary to previous publications which estimated the prevalence of secondary atrial TR at 10%–15% of clinically relevant TRs.^11,12^ However, given the pathophysiology of secondary atrial TR development (annular dilation due to atrial enlargement in patients with atrial fibrillation), and the increasing prevalence of atrial fibrillation, the dominance of secondary atrial TR among other TR aetiologies seems justified and corresponds to everyday clinical practice. Similarly to previous findings, patients with secondary atrial TR in our study were also older, more often women, and more often had atrial fibrillation (including permanent atrial fibrillation) and HF with preserved ejection fraction.^11,12,14^ Despite the oldest age, in-hospital mortality of patients with secondary atrial TR was lower than median in the whole severe TR group.

In our study, 15% of patients were denoted as having predominantly CIED-related aetiology, including 8.9% with CIED-related single aetiology. Previous studies provided conflicting results with prevalence of CIED-related TR ranging from 5% to 26% of patients with severe TR.^15–18^ The results of our study suggest that patients with CIED-related TR are often older, more frail and have signs of more advanced HF compared with other aetiologies of severe TR. Those with CIED-related TR were more often burdened with comorbidities and more often had massive or torrential TR. Patients with CIED-related and secondary ventricular TR were more often hospitalized due to HF decompensation, had more severe HF symptoms, worse left- and right-ventricular function on echocardiography and more often died during hospitalization. Surprisingly, patients with CIED-related TR had even lower LVEF and higher proportion of coexisting HFrEF than those with secondary ventricular TR. Patients with CIED-related TR had worse kidney function and more peripheral oedema, while those with secondary ventricular TR had worse liver function, more often had pleural and pericardial effusion, had highest NT-proBNP concentrations and more often required intravenous diuretic treatment.

The prevalence of primary TR in our study (10%) was consistent with previous reports (5–10%).^1,2,15,19^ Patients with primary TR were the youngest and less often burdened with comorbidities, with the exception of primary mitral regurgitation which was most common in the primary TR group (although still relatively rare). Despite TR most often lasting over 12 months prior to index hospitalization, primary TR was most often associated with less severe TR grade, HF symptoms, lower NT-proBNP concentrations, better kidney function, and better left- and right-ventricular function on echocardiography, less often required diuretic intravenous treatment, and was associated with lower in-hospital mortality compared with other TR groups.

Another important finding of our study is that only 40% of patients with severe TR were evaluated by the Heart Team. That proportion was even lower (26%) for primary and secondary centres, which suggests that organizational reasons and the accessibility to Heart Team evaluation might be partly responsible for this low number. Moreover, it needs to be taken into account that 4.5% of patients died during hospitalization and that at least in 9.4% of patients TR was reduced do moderate or mild (although the latter proportion might potentially be somewhat higher as echocardiography was repeated only in 41% of patients during hospitalization). Still, this leaves ∼86% of all patients with severe TR as potential candidates for Heart Team evaluation, which means Heart Team evaluation should have been performed twice more often. The current ESC guidelines underline the need of timely Heart Team evaluation, which should not be delayed by introduction of aetiology specific and/or symptomatic (i.e. diuretic) treatment.^1^

Only 21% of patients were qualified for an intervention for TR, with the lowest proportion of qualified patients among secondary ventricular TR (14%). This might be attributable to higher risk of those patients, represented by more high-risk characteristics included in TRI-SCORE (more patients in NYHA class III-IV, more pronounced right-sided HF signs with more pleural and pericardial effusion, more need for intensive diuretic treatment, higher creatinine and bilirubin levels, lower LVEF and TAPSE).^20^ However, patients with CIED-related TR were also characterized by worse TRI-SCORE characteristics compared with other TR aetiologies (older age, pronounced right-sided HF signs with more peripheral oedema, lower TAPSE compared with all TR patients, and even higher creatinine levels and even lower LVEF compared with secondary ventricular aetiology). Still, patients with CIED-related TR were more often qualified to interventions than patients with secondary ventricular TR (24% vs 14%), and this included not only lead extractions, but also more frequent qualification to transcatheter tricuspid interventions (17% vs 10%). This might seem surprising given that CIED-related TR is often considered a contraindication to tricuspid TEER. However, multiple studies have shown TEER to be effective for TR reduction in selected patients, especially where CIED-associated mechanism is only one of underlying TR aetiologies.^18,21,22^ Furthermore, other transcatheter procedures, such as orthotopic tricuspid valve replacement or heterotopic bicaval valve implantation, were proven feasible in patients with CIED-associated TR.^16,23,24^

Importantly, in other studies early referral for TR intervention (surgery or transcatheter treatment), especially in those with TRI-SCORE ≤3, was associated with better survival, while in advanced cases (TRI-SCORE ≥6) no survival benefit was observed.^25^ This underlines the need for timely referral of TR patients for intervention, before TR develops into an end-stage, systemic disease. Still, lack of survival benefit in more advanced TR cases does not preclude improvement in symptom control in selected patients.^26,27^

While interpreting in-hospital outcomes, it needs to be emphasized that our study included all patients with severe TR irrespective of the presence of right-sided HF symptoms or the reason for current hospitalization, and that only 40% of patients were hospitalized due to HF decompensation. Overall, in-hospital mortality was 4.5%, with the highest mortality in patients with CIED-related TR (7.4%) and secondary ventricular TR (6.9%). This is not surprising given that those were the patients most often hospitalized for HF decompensation, with more comorbidities, more advanced HF symptoms, worse left- and right-ventricular function, and worse kidney and liver function.

## Limitations

The registry-based character of our study has its limitations, but also advantages, providing real-world estimates of the actual prevalence of different TR aetiologies, as well as characteristics and treatment of patients with severe TR. No additional tests or interventions, apart from those planned by the attending physicians, were performed, and thus, some data on laboratory tests or echocardiography parameters are missing for some patients. However, this strategy enabled waiving the requirement of obtaining signed informed consent from the patients by the ethics committee and thus, inclusion of all consecutive patients with severe TR. Still, for most laboratory and echocardiographic variables, data were available for over 85% of patients, and when the proportion was lower (for bilirubin, right-ventricular dimension, and TAPSE), the table reports the exact percentage of patients for whom the data were available. All other variables were available for all patients. There was no core echocardiographic laboratory in the study, and thus attribution of TR aetiology or severity grade for each patient was done at the discretion of investigators in each participating centre. No data on particular echocardiographic parameters describing TR severity (e.g. effective regurgitant orifice area, regurgitant volume, vena contracta, etc.) or more advanced echocardiographic indices of right-ventricular function (right-ventricular strain, myocardial S’ velocity form tissue Doppler, fractional area change of the right ventricle, or 3-dimensional right-ventricular volumes and ejection fraction) were gathered in the registry. Furthermore, no data from imaging studies other than echocardiography or right-heart catheterization (if performed) were gathered in the registry. However, the relatively low number of variables gathered in the registry, enabled inclusion of a large group of 1295 patients with severe TR.

## Conclusions

In real-life population, unequivocal identification of TR aetiology remained challenging with 1 in 5 patients attributed more than one aetiology. This overlap in aetiologies most often included secondary atrial and ventricular TR, which coexisted in 11% of patients with severe TR. Contrary to previous publications, secondary atrial TR was the most common type of severe TR (37%), followed by secondary ventricular TR (25%). CIED-related and primary TR accounted for 15% and 10% of severe TR, respectively. There were some significant differences in clinical characteristics and outcomes between those aetiology groups, with secondary ventricular and CIED-related TR patients presenting with the most pronounced HF symptoms and burdened with the highest in-hospital mortality. Only 40% of patients underwent evaluation by the Heart Team, and only 21% were qualified for interventions for severe TR, with the lowest proportion of qualified patients among those with secondary ventricular TR.

## Declarations

### Data Availability

No data were generated or analysed for this manuscript.

### Pre-registered Clinical Trial Number

None supplied.
